# Gridded value-added of primary, secondary and tertiary industries in China under Shard Socioeconomic Pathways

**DOI:** 10.1038/s41597-022-01440-0

**Published:** 2022-06-16

**Authors:** Cheng Jing, Buda Su, Jianqing Zhai, Yanjun Wang, Qigen Lin, Miaoni Gao, Shan Jiang, Ziyan Chen, Tong Jiang

**Affiliations:** 1 Collaborative Innovation Center on Forecast and Evaluation of Meteorological Disasters/Institute for Disaster Risk Management/School of Geographical Science, Nanjing University of Information Science & Technology, Nanjing, 210044 China; 2 State Key Laboratory of Desert and Oasis Ecology, Xinjiang Institute of Ecology and Geography, Chinese Academy of Sciences, Urumqi, 830011 China; 3 University of Chinese Academy of Sciences, Beijing, 100049 China; 4 National Climate Center, China Meteorological Administration, Beijing, 100081 China

**Keywords:** Industry, Projection and prediction, Economics

## Abstract

Gridded distribution of future economy plays an important role in climate change impact assessment. The trend of the output values of different industries is crucial for a variety of planning and design processes. Under the Shared Socioeconomic Pathways (SSPs) global framework, the multidimensional model and Cobb-Douglas production model with localized population and economic parameters are used to develop the annual provincial population and value-added of primary, secondary and tertiary industries in China from 2020 to 2100. The most recently implemented fertility-promoting and industrial planning policies in China are considered in our projections. We build multiple models to evaluate the impact of different types of land use on the value-added of primary, secondary and tertiary industries and then gridded the projected value-added to a 5′ × 5′ resolution, based on recorded county-level economic statistics and gridded land use. The reliability of estimations is verified against 2011–2019 statistical data and multiple published datasets. The high-resolution economic dataset is expected to contribute greatly to national and regional climate change impact, adaptation, and vulnerability studies.

## Abstract (summary)

**Table:** 

Measurement(s)	Population • Value-added of industries • Gridded data
Technology Type(s)	multidimensional projection model • Cobb-Douglas production model • computational modeling technique
Sample Characteristic - Location	China

## Background & Summary

Evidence has revealed that the rising concentrations of greenhouse gases due to anthropogenic emissions caused observed global warming^1–4^. Intense economic activities and rapid technological progress have greatly changed the global environment^5^. Moreover, as the warming trend persists, an increasing number of socioeconomic sectors will be exposed to weather and climate extremes globally^6–8^. Socioeconomic patterns under future scenarios have become an important segment in the research realm of climate change impacts and risks^9,10^.

As an essential part of climate projection, climate scenarios provide plausible descriptions of future world conditions and the uncertainty scope under future societal and climate conditions^11,12^. Representative concentration pathways (RCPs), which reflect various concentration targets of greenhouse gases, have been widely applied since the mid-2000s to project potential changes in future climate^13,14^. Thereafter, shared socioeconomic pathways (SSPs) were developed in parallel to characterize future societies facing different mitigation and adaptation challenges^15–17^. As an important complement to previous climate scenarios, SSPs have been widely adopted in the projection of energy, resources, environment and other fields over the last few years^18–22^.

With the signing of the Paris Agreement in 2015, economic development, carbon emissions and global warming have been linked more closer than before^23^. As the second largest economy worldwide, the rapid economic development of China has received global attention. Since the implementation of the reform and opening up policy, China has gradually transitioned into an industrial country from an early agricultural country and is transforming into a service-oriented country at present. According to the national industries classification in China, the primary industry refers to agriculture, forestry, animal husbandry and fishery. The secondary industry largely the manufacturing, mining and construction sectors. The tertiary industry refers to other industries except the primary and secondary industries, includes sectors that serve production and consumption such as commerce, finance, trust, and service industries that provide a variety of labour services. With the decline of agricultural output and the rise of service sector, the contribution of primary, secondary and tertiary industries to the total economic volume is also changing. Nowadays, controlling global warming and carbon emissions has become a common concern for all countries around the world, especially when China announced its carbon neutrality goal. Future changes in the output value of different industrial sectors in China are of great interest^24,25^.

The value-added of an industry, also known as gross domestic product (GDP) by industry, is the contribution of the sector to the overall GDP. As an important part of climate change impact studies, future GDP changes are widely used in losses assessment of weather and climate events such as droughts, floods, wind storm, and tropical cyclones^7,26–28^. Incorporating the economic projection of different industries into the impact assessment can well separate the actual impact of disaster events on specific industries, such as the impact of drought disaster on agriculture, the impact of floods on service industry, and the impact of power shortage caused by heat wave on manufacturing industry. Despite its significance and the demand for accurate economic estimation, research on the future GDP, especially industrial sector-specific research in China, is not sufficient. Near-term projection of the economy is usually based on historical time series via statistical models to extrapolate past economic development trends into the future^29–31^. In comparison, structural economic models based on formal economic theories are more suitable for medium- and long-term economic projections and have been applied by international institutes to project national/regional economies worldwide^32–37^. As a result, future socioeconomic patterns by 2100 under the various SSPs have been released for more than 150 countries^5,38,39^. However, most of the existing long-term economic projections exhibit a coarse spatial resolution at the national level and mainly focus on aggregated outputs. Only a limited number of studies have covered the future development of different industrial sectors, but they have not considered different development pathways^40–42^.

With the development of earth system models and the urgent need for climate risk assessments. the temporal and spatial resolution of climate models is improving^43^. However, corresponding high-resolution socioeconomic data which helps estimate the combined effects of human activities on the carbon-climate system at global and regional scales, are relatively scarce. Risk of climate-related impacts is generally considered to be jointly affected by climate-related hazards with the vulnerability and exposure of human and natural systems^44^. The gridded distribution of future economy will not only directly determine the amount of property exposed to climate events, but is also an important indicator for assessing regional climate vulnerability^45^. people living in region with lower economic levels are more affected and marginalised by climate change and harder to recover from climate change. At the regional scale, high-resolution socioeconomic projections under SSPs are in the early stage of development. In China, key socioeconomic factors, including the population size^8,22,46,47^, Gross Domestic Product^48–51^ and urbanization level^52,53^, have been projected under SSPs based on regional statistics considering newly implemented demographic and economic policies and have been applied to update internationally available projections. Updated regional gridded datasets for China and the Belt and Road region have been widely applied in climate change impact and risk assessments^7,21,26,54,55^. However, it should be noted that a more detailed projection of the population and economic structures is required for the assessment of climate change vulnerability. In this paper, the value-added of primary, secondary and tertiary industries, also referred to as GDP by industries, were projected under five basic SSPs based on localized parameters. And then, a 5′ × 5′ (~85km^2^ around the Equator) resolution gridded dataset for value-added of three industrial sectors in China for the period 2020–2100 is established based on economic projection.

## Data and Methods

**Fig. 1:**
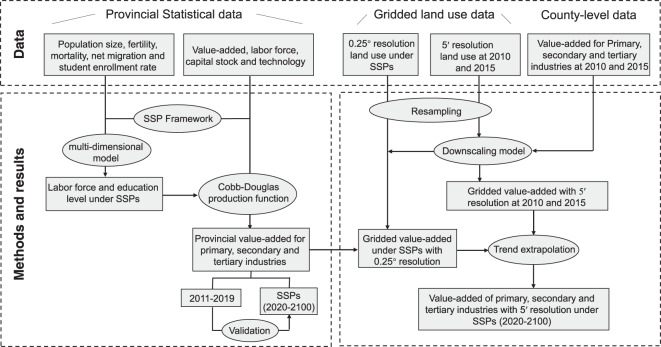
Schematic outline of research process.

As one of the most important factors in economic projection, the population by age, gender and education level in China from 2020 to 2100 needs to be projected first under SSPs. Subsequently, provincial value-added of the primary, secondary and tertiary industries were projected combined with economic statistics. Finally, provincial-level value-added were gridded to a 5′ × 5′ resolution based on recorded county-level value-added and gridded land use. The main process is shown in Fig. 1.

### Data for economic and population projection

#### Demographic data

Demographic censuses are conducted for every 10 years in China, and the sixth demographic census in 2010 was chosen as the initial year to perform population projection. Demographic data including the population size, mortality, net migration by age, gender, educational level, and fertility rate of women by age and educational level in 31 provinces, mainland China, are collected from the Bureau of Statistics of China at http://www.stats.gov.cn/tjsj/pcsj/rkpc/6rp/indexch.htm.

As the main parameter in economic projection, the labour force participation rate (LFPR) and years of schooling from 2000 to 2010 were deduced and divided into two age groups (15–64 and 65+) in the primary, secondary and tertiary industries in 31 provinces based on records retrieved from the fifth and sixth demographic censuses and provincial statistical yearbooks of China (2011).

#### Capital stock

To quantify the capital stock in 2010, we collected the provincial investment and depreciation rate of the capital stock regarding the primary, secondary and tertiary industries from 1978 to 2010. Statistically, gross fixed capital formation could be considered to reflect the annual investment^56,57^. In this study, investment data from 1978–2004 were retrieved from the Data of Gross Domestic Product of China, and data from 2005–2010 were retrieved from the Statistical Yearbook of the Chinese Investment Fixed Assets and provincial statistical yearbooks^58^. The depreciation rate from 1978–2010 was derived by the study of Zong and Liao^59^, who adopted the declining-balance depreciation method to obtain the annual capital depreciation rates of the primary, secondary and tertiary industries based on multiple sets of statistical data. The capital stock of China by province and industry in 1978 was deduced based on the results of Zhang, *et al*.^56^ and applied as main factor to calculate the capital stock in 2010^60,61^.

#### Total factor productivity

The total factor productivity (TFP) and TFP growth rate of the initial year 2010 were applied in the economic projection model. The TFP in 2010 is calculated by the value-added of the industrial sector, capital stock and labour in 2010. The TFP growth rate from 2000–2010 is calculated via the Solow residual method as follows^5,62^:

1 $$GT=GY-\alpha GK-(1-\alpha )GL$$

2 $$\alpha =p\cdot K/Y$$

where *GT* is the TFP growth rate, *GY* is the value-added growth of the different industrial sectors from 2000–2010, which was collected from provincial statistical yearbooks (2001–2011), *GK* and *GL* are the growth rates of the capital stock and labour, respectively, from 2000–2010. α is output elasticity of capital. *p* is the price of capital and set to 0.12. *K* and *Y* are the capital stock and value-added, respectively, in 2010.

### Data for downscaling

#### Gridded land use data

Two sets of land use data were used to grid the projected value-added of the three industries. The historical maps of land use were based on the History Database of the Global Environment (HYDE)^63^. HYDE provides a long-term dataset of historical population estimates and land use reconstructions by assumptions on the trajectory of historical land use per capita. The version of the HYDE 3.2 dataset was used in this paper for establishing the downscaling model with the county scale value-added. Data were provided annually at 5′ spatial resolution from 2000 to 2015. The categories of HYDE 3.2 include cropland (divided into irrigated and rain-fed crops and irrigated and rain-fed rice), grazing lands (divided into more intensively used pasture and less intensively used rangeland) and built-up area. In order to match with the future land use dataset, five land types including cropland, pasture, rangeland, urban (built-up area) and natural vegetation which includes either primary or secondary forest or non-forest (by subtracting the land use, ice, and water fractions from each grid cell) are adopted in this paper.

The land use data under SSPs was generated from the latest CMIP6 (Coupled Model Inter-comparison Project Phase 6) Land Use Harmonization dataset (LUH2)^64^. LUH2 developed a new harmonized set of global land use products based on Integrated Assessment Model (IAM) under eight future projection scenarios (SSP1-1.9, SSP1-2.6, SSP2-4.5, SSP3-7.0, SSP4-3.4, SSP4-6.0, SSP5-3.4-OS and SSP5-8.5) for the time period 850-2100 at 0.25° resolution. Among them, SSP1-2.6, SSP2-4.5, SSP3-7.0, SSP4-3.4 and SSP5-8.5 were used as standard socio-economic scenarios to downscaled the projected provincial value-added under the SSP1-5 respectively for the time period of 2020-2100. The creation of the LUH2 was mainly based on the land use patterns of HYDE 3.2 historical dataset. There are 12 possible land-use states in LUH2, including separation of Primary and Secondary natural vegetation into Forest and Non-forest sub-types, Pasture into Managed Pasture and Rangeland, and Cropland into multiple crop functional types.

#### County-level economic data

County-level economic data, including the value-added of the primary, secondary and tertiary industries in 2010 and 2015 for 2352 counties in the 31 provinces of mainland China, are obtained from the China Statistical Yearbook (county-level) and applied to grid the industrial value-added in 2010 and 2015.

### Data for validation

#### Provincial value-added

Value-added of primary, secondary and tertiary industries from 2011–2019 for the 31 provinces of China are collected from provincial statistical yearbooks (2012–2020) to verify the estimated results from the Cobb-Douglas production function and determine the applicability of the economic projection model in China. In addition, consumer price index (CPI) data from 2005–2019 are collected from the Bureau of Statistics of China to standardize the value-added in the different years.

#### Gridded GDP data

Two sets of gridded GDP data are collected to validate the downscaled results in this study. The first dataset is collected from the RESDC for 2010 and 2015, which exhibits a spatial resolution of 1 km and was established considering the land use type, night light brightness and residential density. The second dataset is a gridded global dataset of the GDP at a 5 arc-min resolution developed by Kummu, *et al*.^65^, which was based on subnational/national GDP and gridded population data and is available at https://www.nature.com/articles/sdata20184. We extracted 2010 and 2015 data of China for verification purposes.

#### Economic data under SSPs

Economic projections of the International Institute for Applied Systems Analysis (IIASA), Potsdam Institute for Climate Impact Research (PIK) and Organization for Economic Cooperation and Development (OECD) are collected and compared to our results under similar assumptions. The IIASA, PIK and OECD were the first organizations to carry out economic projections under SSPs. Projections for more than 150 countries worldwide have been harmonized via the purchasing power parity (PPP) at the 2005 price and published in https://tntcat.iiasa.ac.at/SspDb/dsd?Action=htmlpage&page=10. We extract the estimated results for China and adopt a 2005 PPP conversion factor together with the national CPI in 2005–2010 to convert them to the 2010 RMB price level. The PPP conversion factor is retrieved from the World Bank database (https://data.worldbank.org/indicator/PA.NUS.PRVT.PP).

### Local implications of the SSPs

Five basic SSPs have been developed by the Intergovernmental Panel on Climate Change (IPCC) to describe major socioeconomic, demographic, technological, political, institutional and other related trends in the future^10^. SSP1 is a sustainability pathway in which technological innovation and high-level international cooperation encourage the world to gradually move towards a more sustainable direction. Under SSP1, China is expected to transition into a low-fertility society, with relatively small population size and limited labour force. The focus of economic development gradually shifts to the improvement of the wellbeing of people. Hence, some economic growth may be sacrificed. However, with the continuous development of science and technology, the utilization level of resources may improve, and the gap between the rich and poor may be reduced. SSP2 represents a moderate pathway. Current demographic, capital, and technological trends are maintained in the future. In China, the population fertility, mortality, migration, and education will all be at moderate levels. Technological development will be rapid but without fundamental breakthroughs. The economic development level will also increase, and the education and health care resources of people will improve relatively in a consistent way. SSP3 is a regional rivalry pathway characterized by slow economic development. Investment in education, science and technology is not expected to grow, and the inequality between regions may persist or worsen. In China, fertility will remain high and the population will grow rapidly. The labour force will be sufficient but will exhibit a comparatively low educational level. Both the urbanization speed and migration rate will be limited. SSP4 is an inequality pathway with large regional differences. Regions with labour-intensive industries and a low technological level will be subject to slow economic development. Both the fertility rate and educational level will remain low. A large gap between the rich and poor will promote population concentration in urban areas, and thus, migration will be at a moderate level. SSP5, a fossil fuel development pathway, is subject to rapid economic development. Driven by industrialization and an emerging economy, science and technology, the human capital will rapidly grow, and the investment in health and education will increase. Human fertility and mortality will be at a low level. With the narrowing of the income gap and gradual opening of the labour market, high migration will occur between regions. Partial parameter assumptions in China under the various SSP schemes are listed in Tables 1 and 2.

**Table 1:** Assumptions by SSPs on key influencing factors on population.

	SSP1	SSP2	SSP3	SSP4	SSP5
Fertility	Low	Medium	High	Low	Low
Mortality	Low	Medium	High	Medium	Low
Migration	Medium	Medium	Low	Medium	High
Education	High	Medium	Low	Low	High

**Table 2:** The initial labor force participation rate and output elasticity on capital in 2010 and their assumptions under SSPs in 2050.

Parameters	Industrial sectors	Initial (2010)	SSP1 (2050)	SSP2 (2050)	SSP3 (2050)	SSP4 (2050)	SSP5 (2050)
labor force participation rate (LFPR)	Primary industry	0.345	0.227	0.227	0.255	0.316	0.215
	Secondary industry	0.183	0.170	0.170	0.190	0.181	0.230
	Tertiary industry	0.208	0.325	0.325	0.237	0.241	0.317
output elasticity on capital (α)	Primary industry	0.096	0.091	0.093	0.084	0.084	0.097
	Secondary industry	0.368	0.346	0.357	0.296	0.321	0.367
	Tertiary industry	0.441	0.415	0.428	0.412	0.385	0.457

### Projection of population under SSPs

Changes in the size and education level of the labour force may greatly affect future GDP growth. Many studies have focused on the estimation of the population in China under SSPs, but at a coarse resolution, regarding China as a whole^66,67^. In this study, a multidimensional projection model is parameterized under the SSP global framework considering regional differences in the population size, age, gender and education. The initial fertility, mortality, migration and education levels are defined according to provincial data considering the latest population policy in China^8,46,47^.

During projection, the population is grouped as different educational levels (no education, primary, secondary and tertiary), and the lower-educational groups are converted into the higher-educational groups according to the enrolment rates. Changes in the population at each educational group are based on fertility, mortality, and migration, as expressed in Eq. (3). Equation (4) describes the method of calculating the newborn population (age = 0).

3 $${P}_{prov,g,e,t+1}={P{\prime} }_{prov,e,g,t}\cdot \left(1-{D}_{prov,e,g,t+1}\right)+{M}_{prov,e,g,t+1}$$

4 $${P}_{prov,e=1,t=0}={\sum }_{e=1}^{4}{\sum }_{t=15}^{49}\,{P}_{prov,e,g=female,t}\cdot {F}_{prov,e,t}$$

where *P* is the population in a certain year, *prov*, *g*, *e* and *t* denote the province, gender, education and age of the population, respectively, *P*′ is the population in the previous year, *D* is the mortality, *M* is the net migration, and *F* is the fertility of childbearing-age women.

Table 1 is demonstrated as follows: regarding fertility, the moderate assumption represents the current level. After implementation of the two-child policy, the total fertility rate in China gradually increased to 1.9 in 2019 but is expected to decrease and stabilize at 1.8 after 2021 under the moderate assumption. The fertility under the low/high assumption is 20% lower/higher than that under the moderate assumption by 2030 and 25% lower/higher than that under the moderate assumption by 2050 and beyond. At the provincial scale, the future changes in fertility are determined according to the change range in China.

In regard to mortality, the moderate assumption indicates that the average life expectancy increases by 2 years every 10 years before 2050 and by 1 year every 10 years after 2050 in each province. Under the low/high assumption, the life expectancy will be 1 year lower/higher than that under the moderate assumption.

In terms of migration, the moderate assumption considers that the provincial net migration remains at the current level. Compared to 2010, the net migration is expected to gradually decrease/increase by 50% before 2025 under the low/high assumption and will remain constant thereafter.

According to the educational level, the population is divided into four groups: no education, primary education, secondary (junior and senior high school) education and tertiary (graduate school and higher) education. The primary school, secondary school and university enrolment rates were 96.2%, 93.1% and 27.4%, respectively, in 2010. Under the high assumption, the school scale in each province will expand year by year and approach the level of the most educated countries worldwide (South Korea or Singapore) before 2050, after which it will remain unchanged. At present, the primary school, secondary school and university enrolment rates are 100%, 99.9% and 78.0%, respectively, in South Korea. The low education assumption holds that the educational level will increase at a low rate, almost maintaining the current enrolment rates at all educational levels. For the moderate assumption, average of the high and the low assumptions are adopted by referring to existing study^8^.

### Projection of economy under SSPs

A widely adopted mathematical model, the Cobb-Douglas production function, which considers technical progress, capital and labour input shown in Eq. (5), is implemented to project the value-added of the three industrial sectors in each province of China^5,68^.

5 $$Y\left(t,p,i\right)=L{\left(t,p,i\right)}^{1-\alpha \left(t,p,i\right)}\cdot T\left(t,p,i\right)\cdot K{\left(t,p,i\right)}^{\alpha \left(t,p,i\right)}$$

where *Y* is the GDP in province *p* and year *t* for industrial sector *i*, *L* is the labour input, which is estimated with Eqs. (6) and (7), α is the output elasticity on capital, *T* is the TFP, and *K* is the capital stock.

6 $$L={\sum }_{q=1,2}H(q)\cdot R(q)\cdot W(q)$$

7 $$H=\exp \left\{0.134\cdot min\left(M,4\right)+0.101\cdot min\left[max\left(M-4,0\right),4\right]+0.068\cdot max\left(M-8\right),\left.0\right)\right\}$$

where *q* is the age grouping parameter used to divide the working-age population into two groups (15–64 and 65+), *H* is the educational level, *R* is the working-age population, *W* is the LFPR, and *M* is the average number of years of schooling.

The future LFPR values of the three industrial sectors are based on the SSP framework. The LFPR (15–64) of the primary, secondary and tertiary industries in China in 2010 was 0.345, 0.183 and 0.208, respectively. Under SSP1, SSP2 and SSP4, LFPR (15–64) is considered to present the status of currently developed countries with values of 0.01, 0.15 and 0.5 for primary, secondary and tertiary industries respectively. However, according to the cultivated land planning policy in China, the LFPR (15–64) of the primary industry should maintain 0.05 or above^51^. Therefore, the LFPR (15–64) values of three industrial sectors are set up to reach 0.05, 0.15 and 0.5, respectively, under both SSP1 and SSP2 in 100 years and under SSP4 in 400 years for every province. SSP3 is a slow development pathway, with a large labour force in the primary industry. It is assumed that the LFPR (15–64) in future at all provinces will converge to that of the moderately developed provincial level with value of 0.12, 0.20 and 0.28, respectively, for three industrial sectors within 100 years. Under oriented towards economic development pathway SSP5, the LFPR (15–64) in the future is set up to that of current first-tier developed provinces with values of the primary, secondary and tertiary industries reach 0.02, 0.30 and 0.48, respectively, in 100 years (Table 2). The LFPR of people older than 65 years was 0.188, 0.008 and 0.012 in 2010, at a relatively high level. Evidence has indicated that with increasing development level, the labour participation rate of elderly individuals will decrease^69^. We consider that the LFPR of individuals older than 65 years will decrease under all five SSPs in China, especially in the primary industry. The LFPR of individuals older than 65 years is set up to 0.01, 0.01 and 0.02 considering the three industrial sectors in 100 years.

The output elasticity of capital (α) reflects the ability of capital transformation into the GDP and was calculated by Eq. (2). Statistics have shown that α considering the total industry in China is 0.393 in the initial year (2010). For the primary, secondary and tertiary industries, it is 0.096, 0.368 and 0.441, respectively. The future α is set by referring to the PIK parameterizations^5^. The change range of α in the three industrial sectors in China is assumed to be consistent with that in the total industry, which will converge to 0.35 within 75, 150 and 75 years, respectively, under SSP1, SSP2 and SSP4. Considering slow economic development under SSP3, α of the primary, secondary and tertiary industries is set to reach the level of the moderately developed provinces within 150 years, which is 0.049, 0.100 and 0.330, respectively. Under SSP5, the α value in the future is set to the current level of the first-tier developed provinces in China, which will be 0.10, 0.36 and 0.54 within 250 years, respectively, for the three industrial sectors (Table 2).

The TFP reflects the technological level. The future TFP value can be calculated with the recursive model expressed in Eq. (8):

8 $$T\left(t+1\right)=\left\{\begin{array}{c}t\cdot \left\{{T}_{L}\left(t+1\right)-\left[{T}_{L}\left(t+1\right)-T\left(t\right)\right]\right\}\cdot {e}^{-t\beta }\cdot {\tau }^{-1}+\left(\tau -t\right)\cdot \left(1+{g}_{T}\right)\cdot \\ T\left(t\right)\cdot {\tau }^{-1}t\le \tau \,max\left\{T\left(t\right),{T}_{L}\left(t+1\right)-\left[{T}_{L}\left(t+1\right)-T(t)\right]\cdot {e}^{-t\beta }\right\}t > \tau \end{array}\right.$$

where *T(t)* is the TFP in year t, *T*_*L*_*(t)* is the TFP of the technological leader (the United States) in year t, *g*_*T*_ is the initial TFP growth rate, which is 4.0%, 3.5% and 3.8% for the primary, secondary and tertiary industries, respectively, in China, *β* is the convergence rate and *τ* is the time required for convergence, which is defined based on the SSP narratives and ranges −0.005~0.01 and 20~75, respectively.

Capital stock is an important factor determining economic development. The widely adopted perpetual inventory approach is applied in this study to estimate the provincial capital stock in China during the historical period, as expressed in Eq. (9)^70^. The future capital stock is calculated via the recursive model expressed in Eq. (10).

9 $$K\left(t+1\right)=I\left(t+1\right)+\left(1-\delta \left(t+1\right)\right)\cdot K(t)$$

where *K* is the capital stock, *I* denotes the investments, and *δ* is the depreciation rate. The capital stock in the initial year of 2010 is quantified based on provincial investment and depreciation rate data for the three industrial sectors from 1978–2010.

10 $$K\left(t\right)={\left(\frac{K(t-1)}{L(t-1)}\right)}^{\frac{1-\alpha (t-1)}{1-\alpha (t)}}\cdot {\left(\frac{\alpha \left(t\right)\cdot T\left(t\right)}{\alpha \left(t-1\right)\cdot T\left(t-1\right)}\right)}^{\frac{1}{1-a\left(t\right)}}\cdot L(t)$$

where *K, L, α* and *T* are the capital stock, labour input, capital output elasticity and TFP, respectively.

### Downscaling of the economic data

The gridded value-added of the primary, secondary and tertiary industries in 2010, 2015 and future period for 2020–2100 under SSPs were set up at a 5′ resolution in this study. In this regard, we first built a downscaled model using the county-level value-added with land use data from both 2010 and 2015. Based on the HYDE 3.2 dataset, the areas of five land types cropland, pasture, rangeland, natural vegetation and urban occupied at each county scale were calculated, that is, a total of 4704 county-level samples were obtained.

The distribution of the industrial sectors is usually closely related to land use and land cover patterns. Cultivated land, forestland, grassland and water areas are usually reserved for agriculture, forestry, animal husbandry and fishery, respectively, in the primary industry. The secondary industry is mostly concentrated in built-up area. The tertiary industry mainly refers to the service industry, which is mostly distributed in urban with densely populated areas, but there is also corresponding service industry output in other areas such as farmland, pasture and rangeland. Here, we use a generalized additive model (GAM) to evaluate the impact of different types of land use on the value-added of primary, secondary and tertiary industries. We put the area of cropland, pasture, rangeland, natural vegetation and urban into the GAM model_1 and fit it nonlinearly with the value-added for primary, secondary and tertiary industries. The fitting function is Poisson, and the degrees of freedom are set not more than 4, which shown in Eq. (11).

11 $$E({Y}_{i,r,t})={b}_{0}+S({C}_{r,t},df)+S({P}_{r,t},df)+S({R}_{r,t},df)+S({N}_{r,t},df)+S({U}_{r,t},df)+{\varepsilon }_{i}$$

Where *E* (*Y*_*i,r,t*_) is the expected value-added of industry *i* in region *r* at time *t*; *b*_0_ is the intercept, S() is a smoother of natural cubic splines, *df* is the degree of freedom, *C*_*r,t*_, *P*_*r,t*_, *R*_*r,t*_, *N*_*r,t*_ and *U*_*r,t*_ are the area of cropland, pasture, rangeland, natural vegetation and urban in region *r* at time *t*, *ε*_*i,t*_ is the model error. We built other two models (model_2 and model_3 simultaneously and selected the best-fitting model as the final model for downscaling county-level value-added to the grids, which is shown in Eqs. (12–13)

12 $$\begin{array}{l}E(Y\_p{r}_{i,r,t})={b}_{0}+S(C\_p{r}_{r,t},df)+S(P\_p{r}_{r,t},df)+S(R\_p{r}_{r,t},df)\\ \,\,\,+\,S(N\_p{r}_{r,t},df)+S(U\_p{r}_{r,t},df)+{\varepsilon }_{i}\end{array}$$

13 $$\begin{array}{l}E(Y\_p{p}_{i,r,t})={b}_{0}+S(C\_p{p}_{r,t},df)+S(P\_p{p}_{r,t},df)+S(R\_p{p}_{r,t},df)\\ \,\,\,+\,S(N\_p{p}_{r,t},df)+S(U\_p{p}_{r,t},df)+{\varepsilon }_{i}\end{array}$$

Where parameters with subscript ‘*_pr*’ represent the value per unit area, parameters with subscript ‘*_pp*’ represent the ratio to the total value of the province where the county is located The fitting results of the three fitting models for primary, secondary and tertiary industries are shown in Table 3. The results show that for the primary industry, model_3 has a higher R-square and deviance explained. For secondary and tertiary industries, models_2 and model_3 have essentially the same explanatory power for the outliers, but model_3 have higher R-square value. so model 3 was finally used to fit the primary, secondary and tertiary industries in GAM model.

**Table 3:** The results of downscaling for three industries.

Industries	Type of models	R^2^	Deviance explained	p-value
Primary industry	Model_1	0.404	46.3%	<0.01
	Model_2	0.298	61.1%	<0.01
	Model_3	0.525	63.3%	<0.01
Secondary industry	Model_1	0.564	64.7%	<0.01
	Model_2	0.254	72.4%	<0.01
	Model_3	0.611	69.2%	<0.01
Tertiary industry	Model_1	0.611	74.4%	<0.01
	Model_2	0.691	85.1%	<0.01
	Model_3	0.798	83.8%	<0.01

We put the gridded land use HYDE 3.2 dataset into the fitted GAM model and calculated the estimates of value-added for primary, secondary and tertiary industries on each grid with 5′ resolution. Here, GAM is only used as a downscaling method, and the estimates cannot be directly used as the value-added of the grid. The results need to be calibrated by using county-level statistics. We obtained a calibration factor for each county by dividing the county-level statistics by the total estimates of the grid within the county. The value-added of each grid was then multiplied by the calibration factor to obtain the final gridded value-added for primary, secondary and tertiary industries with 5′ resolution at 2010 and 2015. The calculated calibration factors are also used in the downscaling of future data.

The fitted GAM model was used to estimate the future gridded value-added of the three industries under SSP1-5, based on the LUH2 dataset. LUH2 has a resolution of 0.25 ° (15′), and each LUH2 grid covers exactly nine HYDE 3.2 grids. To maintain uniformity in the magnitude of the independent variables, we resampled the LUH2 data onto the same 5′ resolution grid as HYDE 3.2 and then the gridded value-added were estimated based on the fitted GAM model. Next, we perform a two-step calibration. The grid estimates were first multiplied by the previously calculated county-level calibration factors, to reduce the downscaling error at the county scale. The gridded values were then calibrated at the provincial level. Similarly, the provincial calibration factors were calculated by dividing the provincial projections by the total estimates of the grid within the province. The value of each grid was then multiplied by the provincial calibration factor in order to keep the gridded values match the provincial projections. Next, the value-added on the nine 5′ grids covered by each 0.25° grid were summed, to obtain the future gridded value-added with 0.25° resolution for primary, secondary and tertiary industries under SSP1-5 from 2020–2100.

Finally, a trend extrapolation method was employed to further downscaled the value-added on the future 0.25° grid to the 5′ grid by extrapolating the current economic distribution pattern, which is simple but efficient and has low requirements for the amount of data^71,72^. The value-added of the three industrial sectors in the future are downscaled with Eq. (14).

14 $${G}_{hr\_a}={G}_{hr\_y2}+{f}_{hr\_y2}\cdot \left({G}_{lr\_a}-{G}_{lr\_y2}\right)$$

where *G*_*hr_a*_ is the gridded value-added with high resolution (5′) of each industry in future year *a*, *G*_*hr_y2*_ is the gridded value-added with high resolution in 2015, *f*_*gird_y2*_ is the grid change factor, which can be determined with Eq. (15), *G*_*lr_a*_ is the gridded value-added with low resolution (0.25°) where the high-resolution grid is located in year *a*, and *G*_*lr_y2*_ is the value-added with low resolution in 2015.

15 $${f}_{hr\_y2}=\left(\frac{{G}_{hr\_y2}-{G}_{hr\_y1}}{{G}_{lr\_y2}-{G}_{lr\_y1}}\right)$$

where *G*_*hr_y1*_ and *G*_*hr_y2*_ are the gridded value-added with high resolution in 2010 and 2015, respectively, and *G*_*lr_y1*_ and *G*_*lr_y2*_ are the value-added with a low resolution where the high-resolution grid is located in 2010 and 2015, respectively.

## Data Records

The annual gridded value-added of the primary, secondary and tertiary industries under the various SSPs for China from 2020 to 2100 and the future spatially explicit GDP maps are all available in the public repository 4TH.ResearchData at: 10.4121/14113706.v2^73^.

**Fig. 2:**
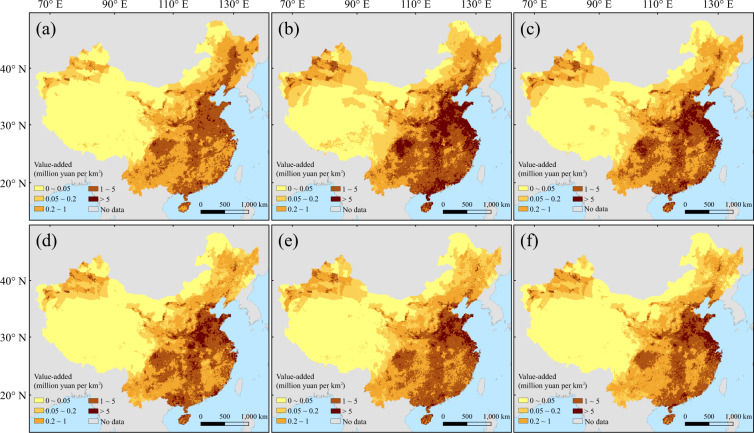
The gridded value-added of primary industry at 2015 (**a**) and 2100 under SSP1-5 (**b**–**f**).

**Fig. 3:**
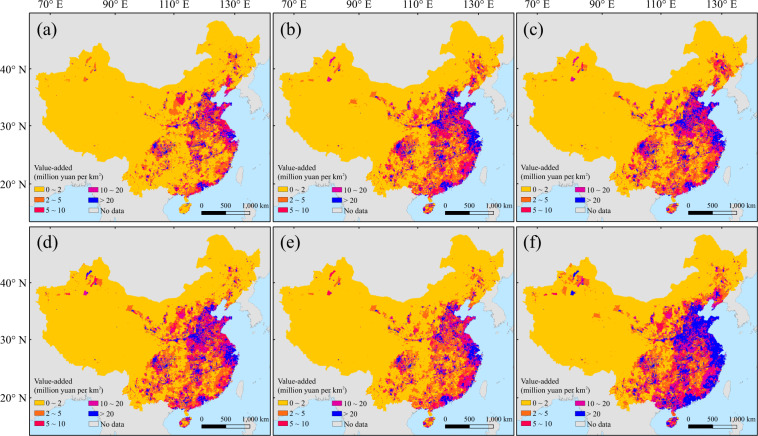
The gridded value-added of secondary industry at 2015 (**a**) and 2100 under SSP1-5 (**b**–**f**).

**Fig. 4:**
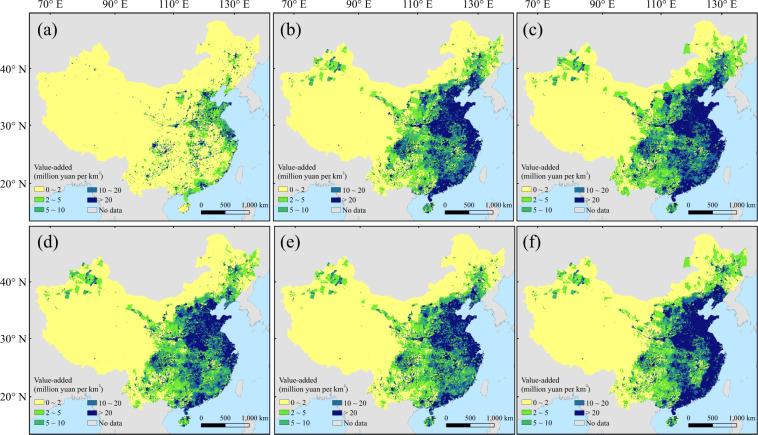
The gridded value-added of tertiary industry at 2015 (**a**) and 2100 under SSP1-5 (**b**–**f**).

The dataset includes value-added at 137996 grids with 5′ (~85km^2^ around the Equator) resolution in mainland China covering 73.50°E-135.00°E and 18.25°N-53.58°N. There are 1215 files in total in 15 compressed packages. Each compressed package is a combination of the grid value-added during 2020–2100 (81 files) for an industry sector and a pathway. The value-added is given as a two-dimensional matrix in units of million yuan. The spatial pattern of changes in value-added of the three industries in 2100 relative to 2015 is shown as an example in Figs. 2–4.

## Technical Validation

### Validation of the economic projections

The year 2010 is considered as initial year in the projection of the provincial-scale industrial value-added. Among all five SSPs, SSP2 maintains the past development trend and is considered as the business-as-usual scenario in research on climate change impacts. Here, we collected economic statistics regarding the three industrial sectors in 31 provinces in China from 2011–2019 to technically validate the deduced industrial value-added during the same time period under SSP2. The Relative error (RE) was used to evaluate the predictive accuracy and bias between estimation and statistics. projection is overestimated when RE is positive, and vice versa.

Table 4 shows the REs in the national and provincial economic projections. For the national estimation, the REs during 2011–2019 are −5.51%~4.73%, −6.85%~2.39% and −3.91%~1.67% for primary, secondary and tertiary industries respectively. our projection is 0.64%, 2.39% and 0.84 less than the statistics for primary, secondary and tertiary industries respectively. For the provincial estimation, the mean errors of all 31 provinces during 2011–2019 are −4.89%~4.80%, −7.62%~4.32% and −4.31%~2.52% for primary, secondary and tertiary industries respectively. Table 5 shows the mean REs during 2011–2019 in each province. Most provinces have relatively low REs, and the absolute value of REs in all provinces and industries are below 9%.

**Table 4:** The relative error between estimated and recorded value-added of the primary, secondary and tertiary industries during 2011–2019.

Region	Industries	Relative error (%)								
		2011	2012	2013	2014	2015	2016	2017	2018	2019
National	Primary industry	−4.45	−5.51	−5.10	−2.81	0.15	1.53	3.40	4.73	2.29
	Secondary industry	−6.59	−6.85	−5.87	−4.60	0.14	2.39	−0.19	−1.19	1.22
	Tertiary industry	−1.50	−0.62	−0.34	1.49	1.67	0.24	−2.58	−3.91	−2.00
Provincial	Primary industry	−3.76	−4.89	−4.60	−2.52	0.94	2.00	4.03	4.80	2.76
	Secondary industry	−7.08	−7.62	−6.25	−4.61	2.26	4.32	0.86	−0.90	1.04
	Tertiary industry	−1.27	−0.50	−0.15	1.82	2.52	0.98	−2.35	−4.31	−2.71

**Table 5:** The mean relative error during 2011–2019 in 31 provinces for the primary, secondary and tertiary industries.

Provinces	Relative error (%)			Provinces	Relative error (%)		
	Primary industry	Secondary industry	Tertiary industry		Primary industry	Secondary industry	Tertiary industry
Beijing	−4.61	0.50	8.04	Hubei	−1.07	−2.80	0.53
Tianjin	−3.39	−8.41	−7.08	Hunan	1.78	−3.67	−0.50
Hebei	−0.41	−5.74	−2.57	Guangdong	1.03	−1.28	−0.34
Shanxi	0.49	−0.16	−2.13	Guangxi	−0.10	−2.20	2.75
Inner Mongolia	−1.73	3.74	−2.61	Hainan	−1.46	−5.93	−0.29
Liaoning	−2.10	−2.41	−2.37	Chongqing	3.03	−2.54	0.48
Jilin	−2.42	−8.95	−7.25	Sichuan	0.59	−3.98	1.30
Heilongjiang	−5.64	−5.11	−6.60	Guizhou	6.05	−1.22	5.61
Shanghai	−6.05	−1.54	−0.14	Yunnan	2.71	0.21	−0.01
Jiangsu	−4.33	−1.75	−3.88	Tibet	6.73	6.93	6.89
Zhejiang	−2.37	−1.03	−1.14	Shaanxi	−0.69	−3.45	−1.51
Anhui	−0.11	−1.38	3.12	Gansu	5.09	−4.62	−2.13
Fujian	0.58	−0.15	1.65	Qinghai	2.40	4.03	−2.28
Jiangxi	−0.22	−2.57	0.75	Ningxia	1.69	−1.85	−0.94
Shandong	−2.65	−3.03	−4.16	Xinjiang	2.00	0.73	−0.92
Henan	0.93	−2.38	−2.86				

**Fig. 5:**
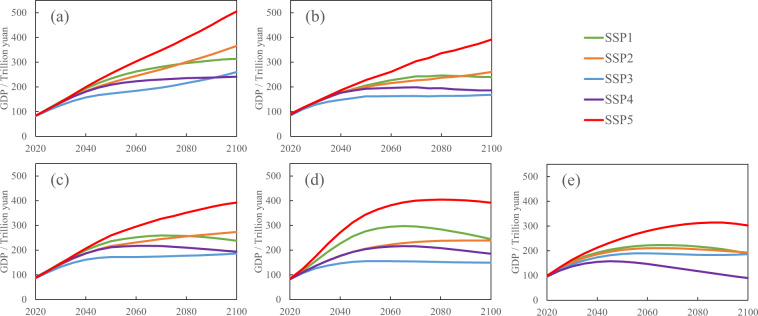
Projected GDP in China under SSPs by ‘two-child’ policy (**a**), by ‘one-child’ policy (**b**), by PIK (**c**), by OECD (**d**) and by IIASA (**e**).

Figure 5 compares the projected GDP levels in China under the various SSPs with the labour force deduced from the population size under the two-child policy in this paper to the results of three international organizations, namely, the PIK, OECD and IIASA. To ensure that the results are comparable, we also projected the GDP under the one-child policy similar to the PIK, OECD and IIASA.

The future GDP in China will increase rapidly until 2050 and then gradually stabilized based on the projections of the PIK and OECD. Moreover, the GDP will reach approximately 400 trillion yuan by the end of the 21st century under SSP5 and ranges from 150~240 trillion yuan under the other four pathways. The GDP projection of the IIASA is lower than that of the other organizations, which is approximately 90~300 trillion by 2100. Under the one-child policy, the projected GDP in this study is basically the same as the PIK result, in which the future GDP in China is much higher under the development-oriented pathway SSP5 than that under the other four pathways. SSP1 and SSP2 simulate a moderate GDP growth with a gradual decrease in the increase rate, while SSP3 and SSP4 simulate a relatively slow growth and even a negative growth, respectively, based on estimates. After adoption of the two-child policy, the GDP in China will maintain a certain growth rate under each pathway. By 2100, the national GDP under SSP1, SSP2, SSP3, SSP4 and SSP5 will reach 314 trillion, 366 trillion, 260 trillion, 241 trillion and 505 trillion yuan, respectively.

### Validation of the data gridding results

**Fig. 6:**
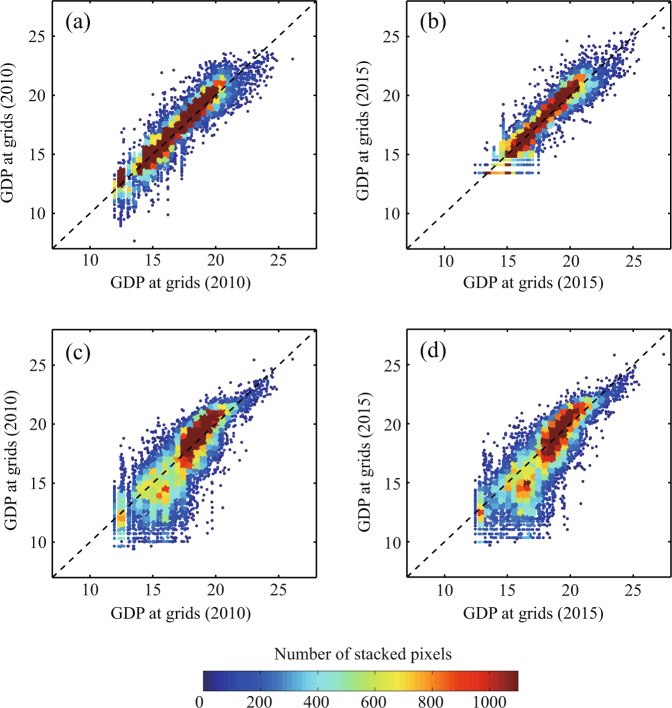
Comparisons of the natural logarithm of gridded GDP in this study with existing datasets of RESDC (**a,b**) and Kummu *et al*. (**c,d**) in 2010 and 2015.

The gridded value-added in 2010 and 2015 determined in this study are compared to the datasets of the RESDC and Kummu *et al*., as shown in Fig. 6. It is clear that our results suitably correlate with the RESDC results, as both gridded datasets are based on county-level records. The RESDC results in some grids are slightly higher than our gridded result, but the majority of the points occur near the centreline. The correlations are 0.86 and 0.89 in 2010 and 2015, respectively, and significant at the 0.05 level. In contrast, the dataset developed by Kummu *et al*., which is gridded based on national and sub-national data, is diverted comparatively larger from our data, but still statistically correlated at the 0.05 significant level with coefficients of 0.77 and 0.72 in 2010 and 2015, respectively. By comparing with the gridded GDP results retrieved from different sources, it was found that the distribution of our gridded data is basically consistent with that reported in existing studies, and our gridding approach is reasonable.

## Usage Notes

We provide a continuous time series of the value-added of the primary, secondary and tertiary industries in China at a 5′ resolution between 2020 and 2100 under five SSPs. There are national and provincial GDP estimation datasets under the SSP framework in China, but the current study is a new attempt to estimate the long-term trend of the value-added of the different industrial sectors at grid scale. We find that the various industries will achieve different development dynamics in the future, and the selection of the development pathway can affect the industrial structure obviously. Moreover, compared to the one-child policy, the new comprehensive two-child policy is likely to promote economic growth.

In recent years, with deepening of climate change impact studies, high-resolution gridded socioeconomic datasets have been required. Scholars have also downscaled regional- and national-scale socioeconomic projections to grids at a constantly improving resolution^65,74–76^, but have focused mainly on gridding the land use and population or GDP during historical periods. As the value-added is mostly documented on an administrative scale, it is difficult to grid. We downscaled the data to 5′ grids, which is performed at a higher resolution than that of most global and regional climate models and is sufficient for future climate change impact studies. Moreover, due to the large number of parameters required for economic projection, our first attempt is carried out in China.

In regard to projections of the population and economy, the initial data are critical to the final outcome. Most of the existing national-scale projections are based on data retrieved from the World Bank or other international agencies^5,38,39^. As our projection is based on officially released regional statistics, including provincial- and county-scale local data, the results are credible. In addition, we correct some of parameters based on relevant literatures. For example, the total fertility rate in China in 2010 was 1.18 according to official records, while a large number of studies have suggested that this rate is an underestimation and should be revised to 1.5^8,77,78^.

There are many available land use data in historical periods, and we have made many modelling attempts^79–81^. Although many data have higher resolution, we finally used HYDE dataset as the basic data to downscaled the value-added. This dataset was combined which historical population estimates and based on improved allocation algorithms to consider the impact of human activities on land change^63^. We have therefore not taken additional population distribution in the downscaling model to avoid possible endogeneity issues. More importantly, this dataset has the same historical land use distribution as the LUH2 used for future downscaling, which makes it more plausible to apply the downscaled model built from the historical data to the future^64^. In addition, the two datasets are spatially compatible, with each LUH2 grid containing exactly nine HUDE3.2 grids, which also makes further downscaling of future results more accessible and tractable.

GAM is used to build the downscaling model because it can fit non-linear parameters and can reflect the amplifying or limiting effects of land size on value-added. GAM is often used as a projection model to obtain the expected value directly based on future changes in the independent variables. In this paper, the using of GAM is not intended to project value-added from land use, but mainly to explore the distribution of different industries’ value-added with land use as a covariate. So the results of the historical estimates must therefore be calibrated at the county level and the calibration coefficients applied to the future to limit excessive deviations in the distribution of value-added due to model errors. The results after the county calibration will also need to be calibrated again for the total provincial values to match the provincial projections. In general, since there are many factors that affect value-added, the model established in this paper cannot be used as a projection model of value-added based on land use, but simply as a downscaling model based on land use distribution pattern.

Datasets on the gridded value-added of the three industrial sectors in China constitute an important breakthrough in the area of SSP-based economic projection. This study fills the gap in current economic projection research, which lacks sector-based estimation and enriches the SSP-based projection database. The results can provide support for the adoption of industrial restructuring measures in China. Additionally, the characteristics of hazard-bearing bodies, which play an important role in climate change exposure, impact and risk assessment studies, can be represented.

## Data Availability

The creation of datasets was done with Matlab R2014b and R-4.1.2. Code used for data preparation and analysis was available at Jing, *et al*.^73^.
